# Ghrelin- and GH-induced insulin resistance: no association with retinol-binding protein-4

**DOI:** 10.1530/EC-13-0019

**Published:** 2013-05-21

**Authors:** Esben Thyssen Vestergaard, Morten B Krag, Morten M Poulsen, Steen B Pedersen, Niels Moller, Jens Otto Lunde Jorgensen, Niels Jessen

**Affiliations:** 1 Medical Research Laboratories Institute of Clinical Medicine, Aarhus University Norrebrogade 44DK-8000, Aarhus C Denmark; 2 Department of Endocrinology and Diabetes Aarhus University Hospital Norrebrogade 44DK-8000, Aarhus C Denmark; 3 Department of Pediatrics Regional Hospital West Jutland Gl. Landevej 61DK-7400, Herning Denmark; 4 Department of Endocrinology and Diabetes Aarhus University Hospital Tage-Hansens Gade 2DK-8000, Aarhus C Denmark

**Keywords:** acyl ghrelin, GH, type 2 diabetes, glucose metabolism

## Abstract

**Objective:**

Supraphysiological levels of ghrelin and GH induce insulin resistance. Serum levels of retinol-binding protein-4 (RBP4) correlate inversely with insulin sensitivity in patients with type 2 diabetes. We aimed to determine whether ghrelin and GH affect RBP4 levels in human subjects.

**Materials and methods:**

To study GH-independent effects of ghrelin, seven hypopituitary men undergoing replacement therapy with GH and hydrocortisone were given ghrelin (5 pmol/kg per min) and saline infusions for 300 min in a randomized, double-blind, placebo-controlled, crossover design. Circulating RBP4 levels were measured at baseline and during a hyperinsulinemic–euglycemic clamp on both study days. To study the direct effects of GH, nine healthy men were treated with GH (2 mg at 2200 h) and placebo for 8 days in a randomized, double-blind, placebo-controlled, crossover study. Serum RBP4 levels were measured before and after treatment, and insulin sensitivity was measured by the hyperinsulinemic–euglycemic clamp technique.

**Results:**

Ghrelin acutely decreased peripheral insulin sensitivity. Serum RBP4 concentrations decreased in response to insulin infusion during the saline experiment (mg/l): 43.2±4.3 (baseline) vs 40.4±4.2 (clamp), *P*<0.001, but this effect was abrogated during ghrelin infusion (mg/l): 42.4±4.5 (baseline) vs 42.9±4.7 (clamp), *P*=0.73. In healthy subjects, serum RBP4 levels were not affected by GH administration (mg/l): 41.7±4.1 (GH) vs 43.8±4.6 (saline), *P*=0.09, although GH induced insulin resistance.

**Conclusions:**

i) Serum RBP4 concentrations decrease in response to hyperinsulinemia, ii) ghrelin abrogates the inhibitory effect of insulin on circulating RBP4 concentrations, and iii) ghrelin as well as GH acutely induces insulin resistance in skeletal muscle without significant changes in circulating RBP4 levels.

## Introduction

GH release from the pituitary gland is considered to be regulated at the hypothalamic level by GH releasing hormone (GHRH) and somatostatin (1). More recently, ghrelin, an endogenous ligand of the GH secretagogue receptor (GHS-R), has been identified in stomach tissue (2). When injected into the systemic circulation, ghrelin is a more potent releaser of GH compared with GHRH (3); in addition to this, ghrelin stimulates ACTH and prolactin secretion (4).

The observation that the GHS-R is also located in peripheral tissues indicates that ghrelin also exerts direct peripheral effects (5, 6). It has recently been reported that exogenous ghrelin causes insulin resistance (7, 8, 9, 10, 11) and induces lipolysis (7, 9, 10, 11, 12, 13). The diabetogenic effects of GH are well established (14, 15), although the exact mechanisms by which GH induces insulin resistance remain to be elucidated.

Retinol-binding protein-4 (RBP4) is a protein and its serum levels are increased in patients with type 2 diabetes and correlate positively with several components of the metabolic syndrome (16). In rodent models, circulating RBP4 correlates positively with insulin resistance and RBP4 directly induces insulin resistance presumably by increased expression of the hepatic gluconeogenic enzyme phosphoenolpyruvate carboxykinase and impaired skeletal muscle insulin signaling (17). Moreover, genetic deletion or reduced serum concentrations of RBP4 by pharmacological remedies increase insulin sensitivity (17). A correlation between insulin sensitivity and serum concentrations of RBP4 has, however, not been consistently reported (18), and a number of reports have questioned the role of RBP4 in insulin resistance (19).

The aim of the present study was to investigate the potential effect of ghrelin- and GH-induced insulin resistance on circulating RBP4 concentrations. To study GH-independent effects of ghrelin, we conducted one experiment in adults with GH deficiency (GHD) receiving an acute i.v. ghrelin infusion; to study direct effects of GH, healthy subjects were treated with GH in a period of 8 days. Insulin sensitivity assessed by the euglycemic clamp technique and measurements of serum RBP4 levels were recorded.

## Materials and methods

### Subjects and experimental design

#### Study 1

Seven otherwise healthy hypopituitary men undergoing stable replacement therapy with GH and hydrocortisone (for >3 months) participated. The diagnosis of GHD was based on an insulin tolerance test (*n*=6) or an arginine stimulation test (*n*=1) with a GH cutoff level ≤3 μg/l. BMI was between 27.5 and 36.0 kg/m^2^ and insulin-like growth factor 1 (IGF1) concentrations were within the normal range.

Each subject was examined twice in a randomized, double-blind, placebo (saline)-controlled, crossover manner. Each study day commenced at 0800 h in a quiet thermoneutral laboratory after 9 h of fasting. The subjects were examined in the supine position and allowed only to drink tap water. One i.v. cannula was placed in the antecubital region for infusions and one was placed in a contralateral dorsal hand vein for arterialized blood sampling.

Preparation of synthetic ghrelin: synthetic human acyl ghrelin (NeoMPS, Strasbourg, France) was dissolved in isotonic saline and sterilized by double passage through a 0.8/0.2 μm pore-size filter (Super Acrodisc, Gelman Sciences, Ann Arbor, MI, USA) by the local hospital pharmacy. From *t*=0 to *t*=300 min, the subjects received a primed continuous ghrelin infusion (5 pmol/kg per min) or a saline infusion. Three of the subjects had subcutaneous (periumbilical) adipose tissue biopsies by liposuction technique at *t*=120 min after applying local analgesic of 10 ml lidocaine (xylocaine 10 mg/ml; AstraZeneca). A hyperinsulinemic–euglycemic clamp (plasma glucose clamped at 5.0 mmol/l, insulin 0.6 mU/kg per min, Actrapid, Novo Nordisk, Copenhagen, Denmark) was performed from *t*=120 to *t*=300 min. The period from *t*=0 to *t*=120 min is referred to as the basal period and the period from *t*=120 to *t*=300 min as the clamp period. Serum RBP4 levels were measured twice each study day: at baseline (*t*=−60 min) and during the clamp at *t*=300 min. Data on glucose metabolism, insulin sensitivity, and energy expenditure from this study have previously been published in a report focusing on the direct metabolic effects of acute ghrelin infusion (11).

#### Study 2

Nine healthy men aged 23.2±0.6 years participated. BMI was 23.1±0.5 kg/m^2^, serum total cholesterol 4.2±0.3 mmol/l, serum LDL 2.2, range 1.5–4.5 mmol/l, serum triglyceride 1.1±0.2 mmol/l, and HbA1c 5.1±0.1%. One subject had mild hypercholesterolemia. None of the participants received any regular medication, and all were nonsmokers.

Each subject was examined immediately before and immediately after an 8-day treatment period with either GH (Norditropin SimplexX; Novo Nordisk; 2 mg s.c. at 1000 h, last injection on day 7) or placebo (saline) injections in a randomized, double-blind, crossover manner. The study periods were separated by a 1- to 3-week washout period.

Participants were prior to the study instructed by a clinical dietician to consume a weight-stable diet containing 50–60% carbohydrates, maximum 30% fat, and protein at 10–15% of total energy intake. Each study day commenced at 0800 h in a quiet, thermoneutral laboratory after an overnight fast. The subjects were examined in the supine position and were allowed only to drink tap water. One i.v. cannula was placed in the antecubital region for infusions and one was placed in a contralateral dorsal hand vein for arterialized blood sampling.

A hyperinsulinemic–euglycemic clamp (plasma glucose clamped at 5.0 mmol/l, insulin 0.6 mU/kg per min, Actrapid) was performed from *t*=200 to *t*=390 min. The period from *t*=0 to *t*=200 min is referred to as the ‘basal period’ and the period from *t*=200 to *t*=390 min as the ‘clamp period.’ Serum RBP4 was measured once each study day at *t*=0.

Data on VLDL kinetics, i.m. triglyceride content, and insulin sensitivity have previously been published in a study focusing on the mechanisms by which GH cause lipolysis and insulin resistance (20).

Both studies were conducted in accordance with the Helsinki Declaration, and all subjects gave their oral and written informed consent to participate. The study protocols were approved by the Local Ethics Committee of Aarhus County, the Danish Medicines Agency and the Good Clinical Practice (GCP) Unit of Aarhus University Hospital.

### Blood samples and measurements

Serum RBP4 levels were measured by an ELISA (EIA) from Alpco Diagnostics (Salem, NH, USA) with an intra-assay coefficient of variation (CV) of 5% and interassay CV of 9.7–9.8%. Plasma glucose was analyzed in duplicate using the glucose oxidase method (Beckman Instruments, Palo Alto, CA, USA). The levels of serum free fatty acid (FFA) were determined using a commercial kit (Wako Chemicals, Neuss, Germany). In study 1, insulin was analyzed with a double monoclonal immunofluorometric assay (Delfia, Perkin Elmer, Wallac Oy, Turku, Finland); in study 2, insulin was determined by commercial ELISA (DAKO, Glostrup, Denmark). IGF1 was measured by a Delfia in-house assay (21). Serum ghrelin (total levels) levels were measured in duplicate by an in-house assay as described previously (22). The assay measures immunoreactive levels of ghrelin using ^125^I-labeled bioactive ghrelin tracer and rabbit polyclonal antibodies raised against octanoylated human ghrelin. The assay recognizes the COOH-terminal of ghrelin and as such determines acylated as well as des-acylated ghrelin. The intra-assay CV averaged 2.8% and samples from each individual were analyzed in one assay.

### Isolation of RNA and real-time RT-PCR

Total RNA was isolated from adipose tissue using TRIzol (Gibco BRL, Life Technologies); RNA was quantified by measuring absorbance at 260 and 280 nm with a ratio ≥1.8 using a NanoDrop 8000 spectrophotometer (Thermo Fisher Scientific, Inc., Waltham, MA, USA). Integrity of the RNA was checked by visual inspection of the two rRNAs, *18S* and *28S*, on an agarose gel. cDNA was synthesized with the Verso cDNA kit AB 1453 (Thermo Fisher Scientific, Inc.) using random hexamers. Real-time PCR for target gene was done with β2-microglobulin levels as internal control, and this expression did not change during intervention. The primers used for PCR are given in Table 1.

The PCRs were performed in duplicate using KAPA SYBR FAST qPCR Kit (Kapa Biosystems, Inc., Woburn, MA, USA) in a LightCycler 480 (Roche Applied Science) using the following protocol: one step at 95 °C for 3 min, then 95 °C for 10 s, 60 °C for 20 s, and 72 °C for 10 s. The increase in fluorescence was measured in real time during the extension step. The relative gene expression was estimated using the default ‘Advanced Relative Quantification’ mode of the software version LCS 480 1.5.0.39 (Roche Applied Science).

### Statistical analysis

Results are expressed as mean±s.e.m. or geometric mean and 25–75% range. Comparisons before and after treatment were carried out by a paired *t*-test or, if non-normal distributed data, with a signed rank test. Correlations were calculated by using Pearson's linear regression coefficient. A *P* value <0.05 was chosen as level of significance. The number of subjects is indicated by *n*. Analyses were performed using SigmaPlot version 11.0 for Windows.

## Results

### Study 1

Serum ghrelin concentrations increased to 5.3±0.5 μg/l in the basal period and 5.9±0.5 μg/l during the clamp period, which was a tenfold increase compared with saline administration. We recorded no concomitant increase in serum concentrations of GH or cortisol. Ghrelin-induced hyperglycemia: 5.9±0.2 mmol/l (ghrelin) vs 5.4±0.1 mmol/l (saline), *P*=0.009, in the basal period and insulin resistance during the clamp period, where ghrelin concentrations significantly reduced the glucose infusion rate (*M* value; mg/kg per min): 1.40±0.47 (ghrelin) vs 3.19±0.58 (saline), *P*<0.001. Serum-FFA concentrations increased (mmol/l) after ghrelin exposure: 0.62±0.03 (ghrelin) vs 0.43±0.07 (saline), *P*=0.047. Ghrelin infusion did not affect endogenous glucose production (data not shown).

#### RBP4

**Figure 1:**
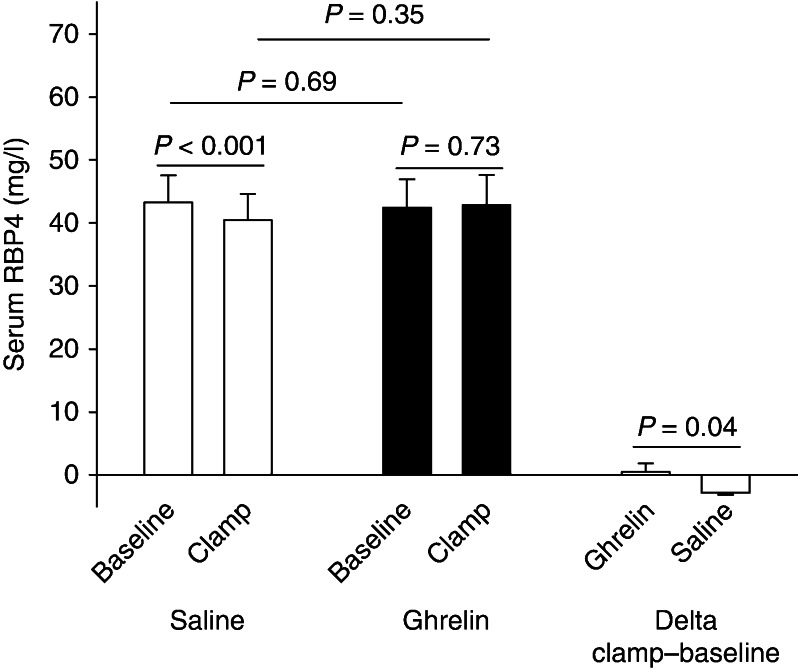
Serum retinol-binding protein-4 (RBP4) concentrations at baseline and in response to hyperinsulinemia and hyperinsulinemia combined with ghrelin infusions in hypopituitary men. Serum RBP4 levels decreased in response to hyperinsulinemia, whereas ghrelin infusion abrogates the inhibitory effect of insulin on circulating RBP4 concentrations. The delta value between RBP4 concentrations at baseline and during hyperinsulinemia was also significantly reduced by concomitant ghrelin infusion.

**Figure 2:**
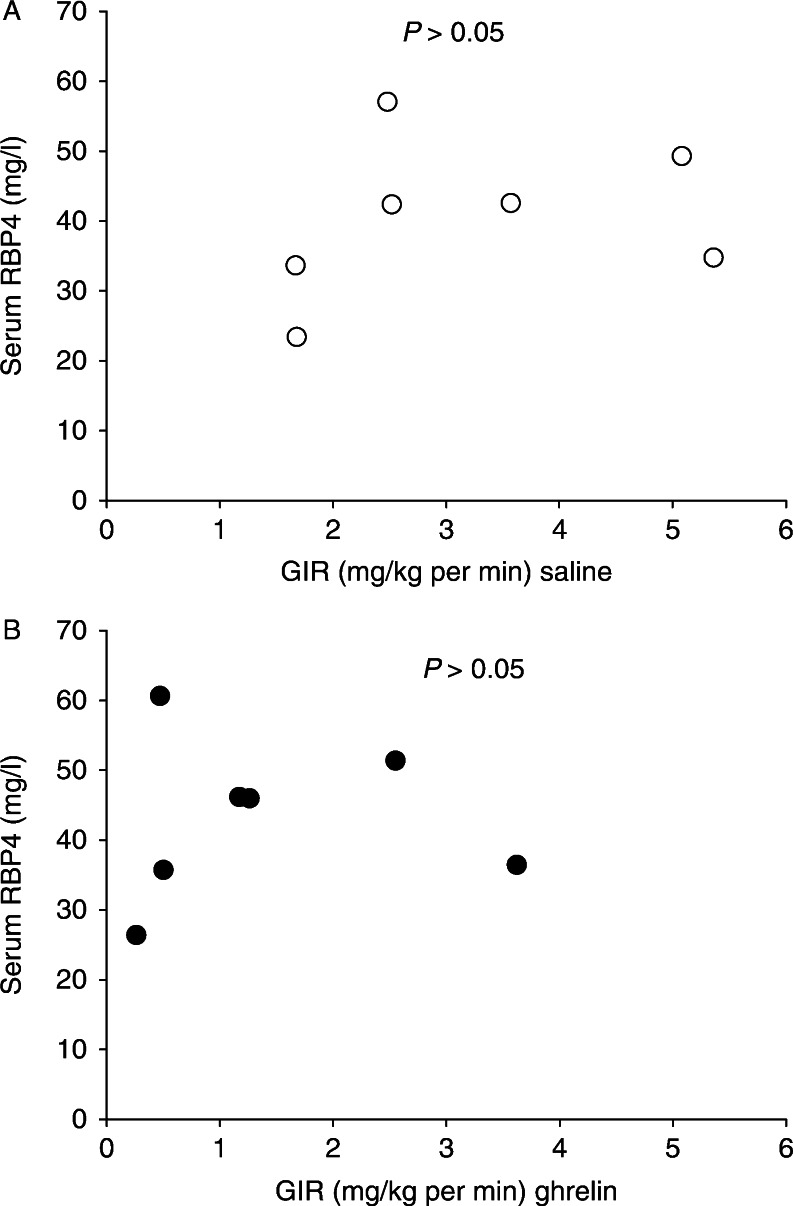
Serum retinol-binding protein-4 (RBP4) concentrations and corresponding glucose infusion rates (GIR) during hyperinsulinemia (A) and during hyperinsulinemia and ghrelin infusion (B) in hypopituitary men. There was no statistical linear correlation between these variables. Open circle, hyperinsulinemia without ghrelin; filled circle, hyperinsulinemia and ghrelin.

Baseline serum levels of RBP4 (mg/l) were similar on both study days (42.4±4.5 (ghrelin) vs 43.2±4.3 (saline), *P*=0.69). Serum RBP4 (mg/l) concentrations decreased in response to insulin infusion in the saline study (43.2±4.3 (baseline) vs 40.4±4.2 (clamp), *P*<0.001), whereas concomitant ghrelin infusion abrogated this suppressive effect of insulin on RBP4 (mg/l; 42.4±4.5 (baseline) vs 42.9±4.7 (clamp), *P*=0.73; Fig. 1). There was no effect of ghrelin on serum concentrations of RBP4 during the clamp period (mg/l; 42.9±4.7 (ghrelin) vs 40.4±4.2 (saline), *P*=0.35; Fig. 1), and there was no correlation between insulin sensitivity and RBP4 levels, *P*>0.05 (Fig. 2). In particular, the change in RBP4 levels between the basal period and the clamp period (ΔRBP4) did not correlate significantly with insulin sensitivity as assessed by the *M* value (*r* ^2^=0.21, *P*=0.27).

#### Gene expressions

RBP4 levels in adipose tissue were increased during ghrelin administration as follows: subject 1, 27%; subject 2, 260%; and subject 5, 62% corresponding to an average increase of 117% in relation to the gene expression level during the saline experiment.

### Study 2

**Figure 3:**
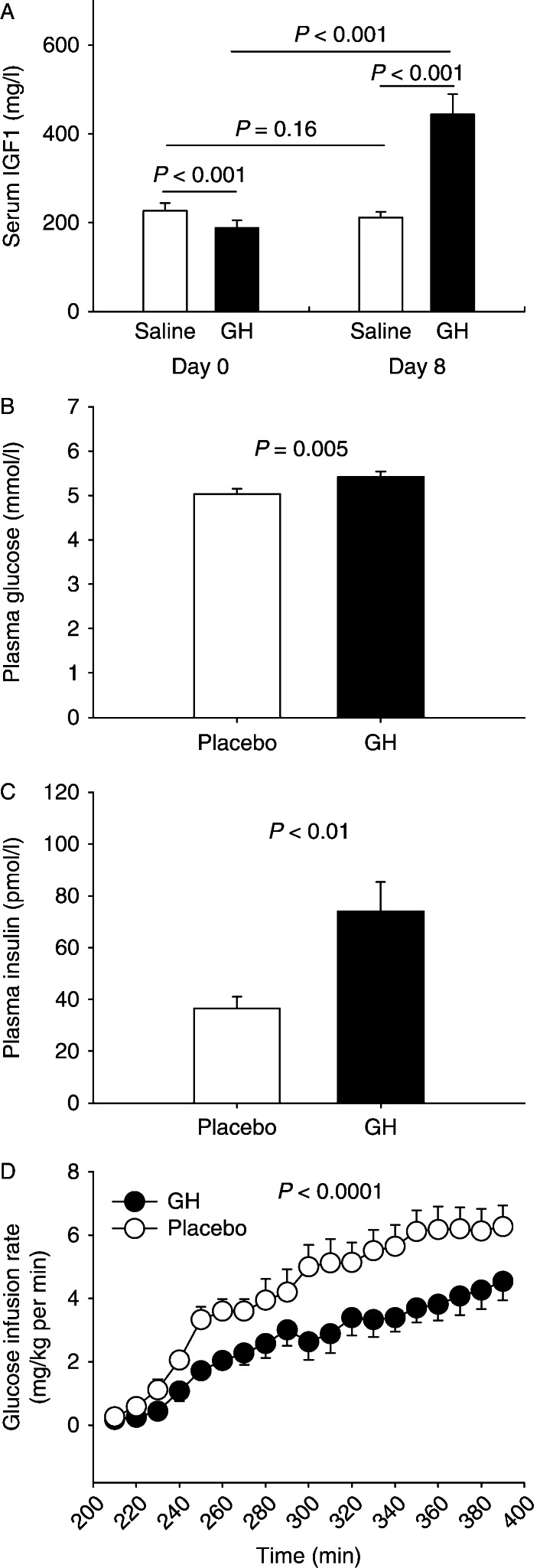
Serum insulin-like growth factor 1 (IGF1) concentrations in healthy young men at baseline and after 8 days of placebo and GH administration respectively. GH induced a significant increase in IGF1 concentrations (A). Basal fasting plasma glucose (B) and plasma insulin (C) concentrations were significantly increased after 8 days of GH administration compared with 8 days of placebo administration. Insulin sensitivity was significantly impaired by 8 days of GH administration compared with 8 days of placebo administration (D). Glucose infusion rates (GIR) indicated by open circle during hyperinsulinemia after 8 days of placebo administration and by filled circle during hyperinsulinemia after 8 days of GH administration.

As expected, serum IGF1 concentrations increased significantly after 8 days of GH treatment compared with baseline (Fig. 3A). GH treatment increased fasting plasma glucose (mmol/l): 5.4±0.1 (GH) vs 5.0±0.2 (saline), *P*=0.008 (Fig. 3B) and fasting serum insulin (pmol/l, median (range)): 52.0 (41–138) (GH) vs 25.5 (15.5–54) (saline), *P*<0.01 (Fig. 3C). The *M* value during hyperglycemia was reduced by GH treatment (mg/kg per min): 4.1±0.6 (GH) vs 6.2±0.7 (saline), *P*<0.0001 (Fig. 3D).

#### RBP4

**Figure 4:**
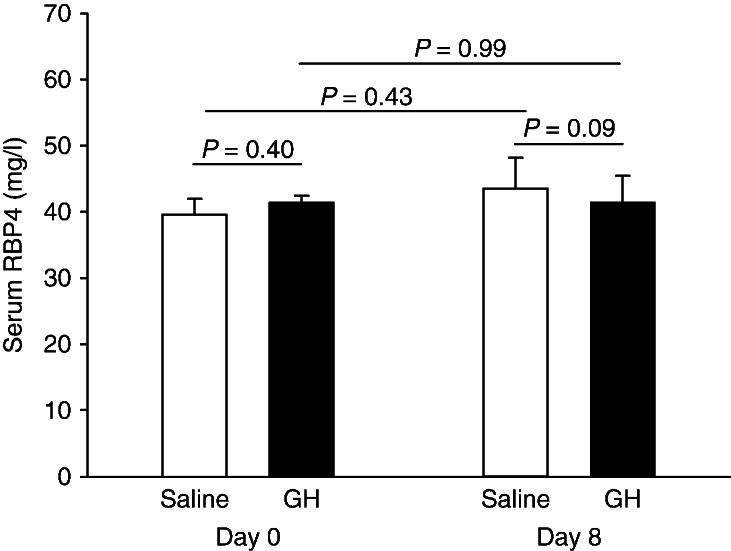
Serum retinol-binding protein-4 (RBP4) concentrations in healthy young men at baseline and after 8 days of placebo and GH administration respectively. GH did not impact on serum RBP4 concentrations.

**Figure 5:**
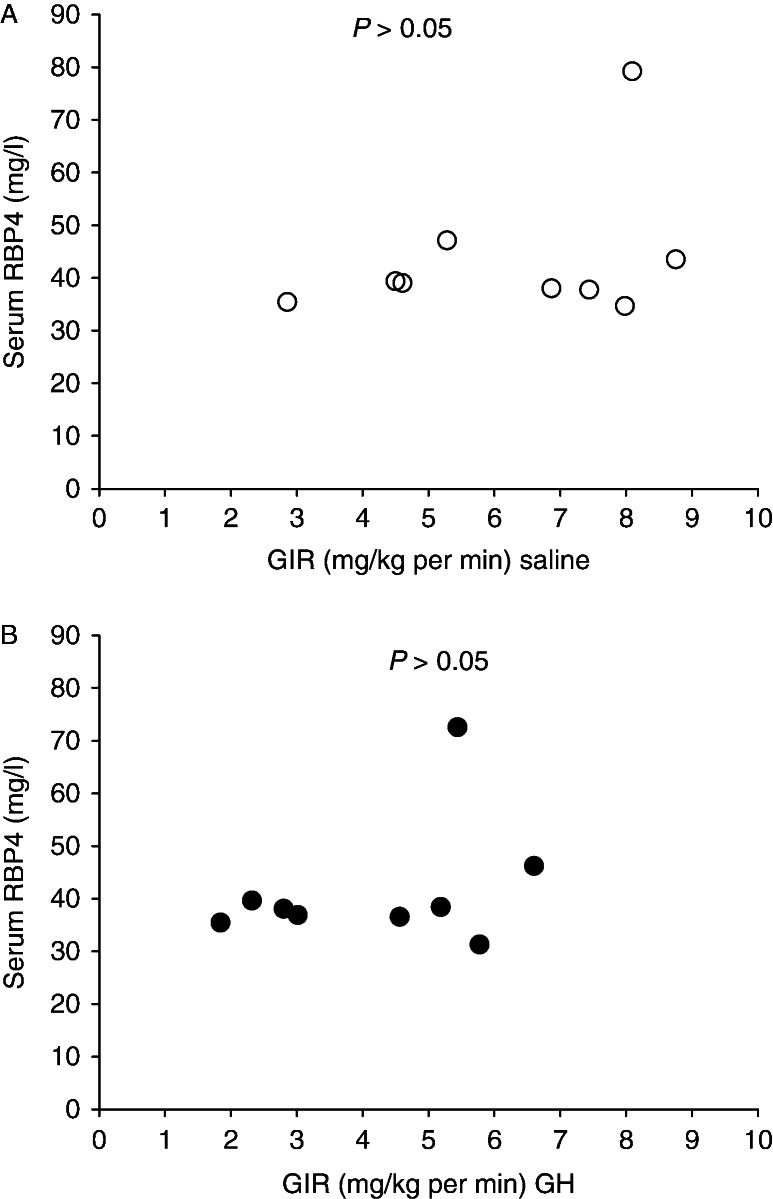
Serum retinol-binding protein-4 (RBP4) concentrations and corresponding glucose infusion rates (GIR) during hyperinsulinemia after placebo treatment (A) and during hyperinsulinemia after 8 days of GH administration (B) in healthy young men. There was no statistical linear correlation between these variables. Open circle, hyperinsulinemia after 8 days of placebo administration; filled circle hyperinsulinemia after 8 days of GH administration.

Serum RBP4 concentrations were similar before the interventions (mg/l): 41.6±1.1 (GH) vs 40.0±2.4 (saline), *P*=0.40, and also after the interventions (mg/l): 41.7±4.1 (GH) vs 43.8±4.6 (saline), *P*=0.09 (Fig. 4). There was no correlation between insulin sensitivity and serum RBP4 concentrations after GH administration or after saline administration, *P*>0.05 (Fig. 5).

## Discussion

Here, we report that insulin resistance induced by either ghrelin infusion in hypopituitary men or GH treatment in healthy young men is not associated with serum concentrations of RBP4. RBP4 is an adipokine reported to directly induce insulin resistance, whereas insulin sensitivity is improved by either genetic deletion of the *RBP4* gene or lowering of serum concentrations of RBP4 (17). Yang *et al*. (17) first described that *Glut4* ^*−/−*^ (*Slc2a4* ^*−/−*^) insulin-resistant mice had increased expression of *Rbp4* gene in adipose tissue and that the PPARγ agonist rosiglitazone, a well-known insulin sensitizer, reduced *Rbp4* expression. They also described that skeletal muscle PI3K activity, considered to be a rate-limiting step in the insulin-signaling cascade, significantly decreases in the presence of excessive RBP4 levels in *Rbp4* overexpression transgenic mice (17). So, in rodents, RBP4 is closely linked to insulin resistance. RBP4 is considered to be secreted from adipocytes in response to declining blood glucose levels and to cause insulin resistance and thereby counter regulate hypoglycemia. In support of this, RBP4 production is reported in human adipocytes (23) and RBP4 inhibits activation of IRS-1, a proximal enzyme in the insulin-signaling cascade, *in vitro* (24). In one clinical trial, serum RBP4 levels correlated with insulin resistance (in obese subjects) and impaired glucose tolerance or type 2 diabetes in nonobese subjects with a family disposition to type 2 diabetes (25). In healthy human subjects, data on the association between RBP4 levels and insulin sensitivity are divergent: Promintzer *et al*. (18) investigated whether there was an association between RBP4 levels and insulin sensitivity in healthy human subjects and they did not record any association. Ribel-Madsen *et al*. (26) did a large study in healthy subjects and used hyperinsulinemic–euglycemic clamps to investigate the association between RBP4 levels and insulin sensitivity. They reported that RBP4 correlated inversely with glucose rate of disappearance, but the association disappeared after adjusting for differences in adiponectin levels indicating that RBP4 is not a key regulator of peripheral glucose uptake. Kowalska *et al*. (27) investigated healthy lean and obese women with normal glucose tolerance and demonstrated an inverse correlation between RBP4 and glucose rate of disappearance independent of potential confounders. The reason for these discrepancies has not been established, but the potential association between circulating RBP4 and insulin sensitivity warrants further investigations and is the basis for our present study although the basis for expecting changes in circulating RBP4 by acute ghrelin or GH infusion is lacking currently.

Injection of ghrelin in human subjects elicits dose-dependent GH and cortisol secretion (4), and infusion of ghrelin also increases plasma levels of glucose and FFAs (7, 9, 10, 12) and induces peripheral insulin resistance (8, 10). We have earlier investigated the GHS potency and diabetogenic effects of ghrelin infusions in healthy subjects without (9) and with concomitant somatostatin infusion (10) and reported that ghrelin induced GH secretion and insulin resistance in both settings. In the present study, we aimed to investigate the potential isolated effect of ghrelin and GH on serum RBP4 levels and therefore did not investigate the effect of ghrelin on RBP4 levels in healthy subjects. In the present study comprising hypopituitary subjects, ghrelin infusion did not result in GH or cortisol secretion (11). This demonstrates that the observed suppression of insulin-stimulated glucose disposal in skeletal muscle and increased FFA turnover were attributable to ghrelin infusion, whereas ghrelin did not affect hepatic insulin sensitivity (11) and RBP4 was not associated with ghrelin-induced insulin resistance. In a more recent report, we confirmed that ghrelin directly causes lipolysis in peripheral tissues (13). The mechanisms by which ghrelin induces insulin resistance and lipolysis remain to be investigated, and in this report, we have focused on the potential role of RBP4 in ghrelin-induced insulin resistance. Analyses of the adipose tissue by real-time PCR technique from the three patients, who volunteered to have fat biopsies taken, showed an association between ghrelin and *RBP4* gene expression in adipose tissue. To investigate whether this association was due to a putative receptor-mediated effect of ghrelin on *RBP4* gene expression in adipocytes, we performed a pilot study where we incubated human adipose tissue±ghrelin for 48 h *in vitro*. We did not see any regulation of *RBP4* mRNA under these circumstances, indicating that the increase in *RBP4* gene expression *in vivo* could be an indirect effect of ghrelin. One suggestion is that the *in vivo* effect of ghrelin could be mediated through increased sympathetic nervous system activity (28), but more mechanistic studies are needed to fully elucidate this connection and this was not considered within the scope of the present study. Thus, acute ghrelin infusion upregulates *RBP4* gene expression in adipose tissue, but this does not translate into measurable alterations in circulating RBP4 concentrations, suggesting that RBP4 is not involved in ghrelin-induced insulin resistance.

In study 1 comprising hypopituitary subjects, we recorded a significant reduction of serum RBP4 concentrations in response to hyperinsulinemia during saline administration, and this observation is in line with the observations by Promintzer *et al*. (18) who reported a decrease of plasma RBP4 in response to a hyperinsulinemic–euglycemic clamp in overweight healthy insulin-sensitive as well as insulin-resistant adults. It has been hypothesized that decreased glucose uptake by adipocytes may stimulate RBP4 secretion as a counterregulatory response (17). If this mechanism is physiologically relevant, our observation could be explained by increased glucose uptake in adipocytes during hyperinsulinemia in the saline experiment and decreased glucose uptake in adipocytes during hyperinsulinemia in the ghrelin experiment, leading to i) a compensatory reduction of RBP4 secretion in the saline experiment and ii) no change in RBP4 secretion in the ghrelin experiment. Another hypothesis to explain this observation is that ghrelin infusion stimulates adipose tissue *RBP4* gene expression (as indicated by the three adipose tissue biopsies) and RBP4 secretion, and this stimulating effect of ghrelin counteracts the inhibitory effect of insulin on serum RBP4 concentrations during saline infusion.

In the study comprising the healthy controls, insulin resistance was induced by pharmacological GH doses. The metabolic effects of GH have been recognized for decades (15) and GH signaling has recently been documented in human peripheral target tissues *in vivo* (29). The molecular mechanisms whereby GH induces insulin resistance in skeletal muscle, however, remain uncertain, and it is dubious whether they include distinct suppression of insulin signaling (30). In the present study, circulating RBP4 concentrations were not associated with GH-induced insulin resistance, suggesting that RBP4 does also not play a major role in GH-related changes of glucose homeostasis.

The strength of our two clinical studies is the crossover design where each subject is examined twice with and without ghrelin and GH respectively. The weakness of our study is the limited number of patients and healthy controls and, hence, the risk of making a type 2 error. There seems, however, not to be any effect of either ghrelin or GH on RBP4 levels at all. In both the ghrelin and the GH studies, insulin resistance was caused by reduced peripheral insulin sensitivity whereas insulin sensitivity of the liver remained unaffected. It is therefore likely that hepatic rather than peripheral insulin resistance interacts with RBP4.

In conclusion, these clinical studies do not support a causal association between insulin resistance and RBP4 serum levels in human subjects. The observed suppression of RBP4 levels in response to a hyperinsulinemic–euglycemic glucose clamp merits further investigation.

## Author contribution statement

E T Vestergaard was involved in the protocol, clinical trial, data analysis, discussion of results, manuscript first draft, and manuscript revision. M B Krag was involved in the protocol, clinical trial, data analysis, discussion of results, and manuscript revision. M M Poulsen, S B Pedersen, and N Jessen were involved in the PCR analyses, data analysis, discussion of results, and manuscript revision. N Moller and J O L Jorgensen were involved in the protocol, data analysis, discussion of results, and manuscript revision.

## Figures and Tables

**Table 1:** Primers used for real-time PCR analysis

**Gene**	**Sense** (5′–3′)	**Antisense** (5′–3′)
RBP4	GAC AAC ATC GTC GCG GAG TT	CCA TGT CTG CGC ACA CGT CCC
β2-microglobulin	GAG GCT ATC CAG CGT ACT CC	AAT GTC GGA TGG ATG AAA CCC
